# An interpretable machine learning model for predicting in-hospital mortality in ICU patients with ventilator-associated pneumonia

**DOI:** 10.1371/journal.pone.0316526

**Published:** 2025-01-07

**Authors:** Junying Wei, Heshan Cao, Mingling Peng, Yinzhou Zhang, Sibei Li, Wuhua Ma, Yuhui Li

**Affiliations:** 1 First Clinical Medical College, Guangzhou University of Chinese Medicine, Guangzhou, Guangdong, China; 2 Department of Neurology, Sun Yat-sen Memorial Hospital, Sun Yat-sen University, Guangzhou, Guangdong, China; 3 Department of Anesthesiology, Shanghai General Hospital, Shanghai Jiao Tong University, Shanghai, China; 4 Department of Anesthesiology, The Third Affiliated Hospital, Sun Yat-sen University, Guangzhou, Guangdong, China; 5 Department of Anesthesiology, The First Affiliated Hospital of Guangzhou University of Chinese Medicine, Guangzhou, Guangdong, China; Children’s National Hospital, George Washington University, UNITED STATES OF AMERICA

## Abstract

**Background:**

Ventilator-associated pneumonia (VAP) is a common nosocomial infection in ICU, significantly associated with poor outcomes. However, there is currently a lack of reliable and interpretable tools for assessing the risk of in-hospital mortality in VAP patients. This study aims to develop an interpretable machine learning (ML) prediction model to enhance the assessment of in-hospital mortality risk in VAP patients.

**Methods:**

This study extracted VAP patient data from versions 2.2 and 3.1 of the MIMIC-IV database, using version 2.2 for model training and validation, and version 3.1 for external testing. Feature selection was conducted using the Boruta algorithm, and 14 ML models were constructed. The optimal model was identified based on the area under the receiver operating characteristic curve (AUROC), accuracy, sensitivity, and specificity across both validation and test cohorts. SHapley Additive exPlanations (SHAP) analysis was applied for global and local interpretability.

**Results:**

A total of 1,894 VAP patients were included, with 12 features ultimately selected for model construction: 24-hour urine output, blood urea nitrogen, age, diastolic blood pressure, platelet count, anion gap, body temperature, bicarbonate level, sodium level, body mass index, and whether combined with congestive heart failure and cerebrovascular disease. The random forest (RF) model showed the best performance, achieving an AUC of 0.780 in internal validation and 0.724 in external testing, outperforming other ML models and common clinical scoring systems.

**Conclusion:**

The RF model demonstrated robust and reliable performance in predicting in-hospital mortality risk for VAP patients. The developed online tool can assist clinicians in efficiently assessing VAP in-hospital mortality risk, supporting clinical decision-making.

## Introduction

Ventilator-associated pneumonia (VAP) is the predominant hospital-acquired pneumonia among patients undergoing mechanical ventilation (MV) in Intensive Care Units (ICU) [1]. Recent studies have demonstrated that among ICU patients on MV, VAP incidence can reach 24% -39% [2], with mortality rates spanning 25% to 50% [3]. VAP significantly burdens the healthcare system, resulting in increased healthcare expenditures, longer ICU stays, and higher ICU mortality [4]. Hence, the development and deployment of precise and reliable clinical tools for assessing mortality risk in VAP patients not only plays an important role in facilitating early clinical decisions but also in the reasonable allocation of existing medical resources [5].

In clinical practice, numerous disease severity scoring systems, such as the Acute Physiology Score (APS III), Simplified Acute Physiology Score (SAPS II), Logistic Organ Dysfunction System (LODS), and Oxford Acute Severity of Illness Score (OASIS), are widely used for risk assessment of ICU patients [3]. However, commonly used scoring systems like APS III and SAPS II, due to their complexity and time-consuming calculations, impose an additional burden on clinical operations. Moreover, the specificity of these scoring systems is often suboptimal, as they primarily evaluate the overall severity of ICU patients’ conditions and may not effectively predict mortality risk in patients with VAP.

In recent years, the rapid advancement of artificial intelligence (AI), particularly machine learning (ML), has garnered considerable interest for its substantial potential in clinical applications, progressively transforming critical care medicine and advancing the development of precision medicine [6, 7]. A meta-analysis has shown that various ML techniques have been employed to develop early prediction models for VAP, with most demonstrating promising predictive performance [8]. Nevertheless, there remains a paucity of predictive models specifically addressing in-hospital mortality risk among VAP patients [9].

This study aims to develop an effective ML model for predicting in-hospital mortality risk in patients with VAP based on the Medical Information Mart for Intensive Care IV (MIMIC-IV) database and validate the model’s generalizability through external testing. Furthermore, SHapley Additive exPlanation (SHAP) was employed to generate interpretable visualizations of predictive outcomes, and a web-based tool was developed to facilitate efficient risk assessment, enabling healthcare professionals to make more effective clinical decisions.

## Materials and methods

### Study population

The data for this retrospective study were sourced from the MIMIC-IV database (versions 2.2 and 3.1) [10, 11]. The MIMIC-IV database is an open-access, large-scale critical care database organized and maintained by the Laboratory for Computational Physiology at the Massachusetts Institute of Technology (MIT). Version 2.2 comprises healthcare data for nearly 300,000 patients treated at the Beth Israel Deaconess Medical Center (BIDMC) from 2008 to 2019, which was used for model training and internal validation. Version 3.1 includes additional healthcare data from 2020 to 2022, which was employed for external testing of the model (S1 Dataset). Ethical approval for the use of MIMIC-IV data was granted by the Institutional Review Boards of both BIDMC and MIT. All personal data within the database were de-identified, thus waiving the need for informed consent. The author (Junying Wei) has been granted access to the dataset (certification number: 62985887). The reporting of this study adhered to the Transparent Reporting of a Multivariable Prediction Model for Individual Prognosis or Diagnosis—AI (TRIPOD-AI) guidelines [12] (S1 Table).

**Fig 1:**
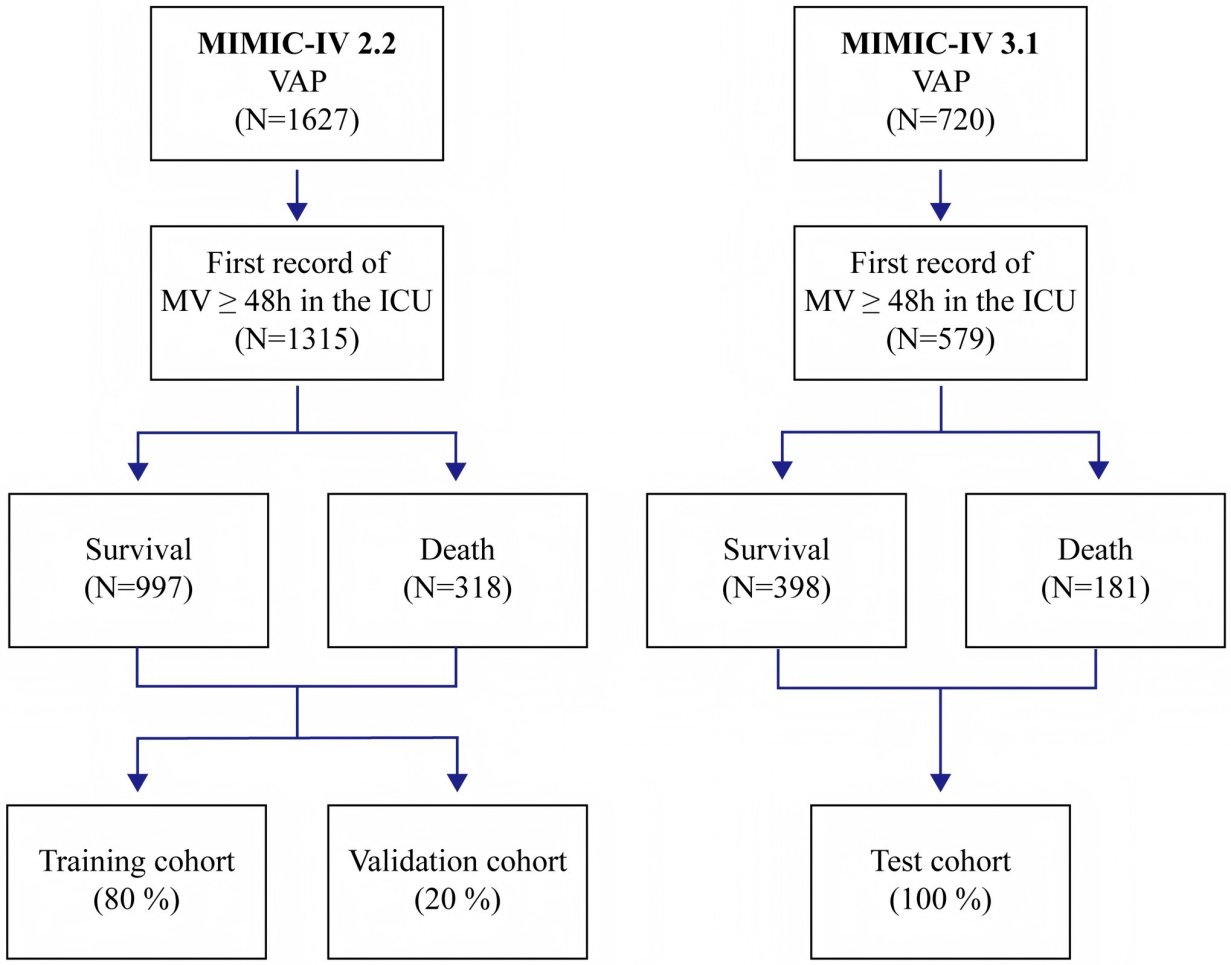
Inclusion and exclusion flowchart.

Patients with VAP in the MIMIC-IV database were identified based on International Classification of Diseases, Ninth or Tenth Revision (ICD-9 or ICD-10) codes. The ICD codes for VAP are 99731 (ICD-9) and J95851 (ICD-10). Records were included only if the initial duration of MV was 48 hours or longer. The inclusion and exclusion criteria are outlined in Fig 1.

### Data collection and processing

Structured Query Language (SQL) was used to extract variables from the MIMIC-IV database, including demographics, vital signs, laboratory indicators, comorbidities, clinical scores, and survival status during hospitalization. Vital signs, laboratory indicators were recorded within 24 hours following the initial 48 hours of MV. For indicators with multiple records, the average value was taken. Features with a missing rate exceeding 20% were excluded to mitigate bias resulting from incomplete data. Other features with a missing rate within 20% were handled using multiple imputation (S1 Fig).

**Fig 2:**
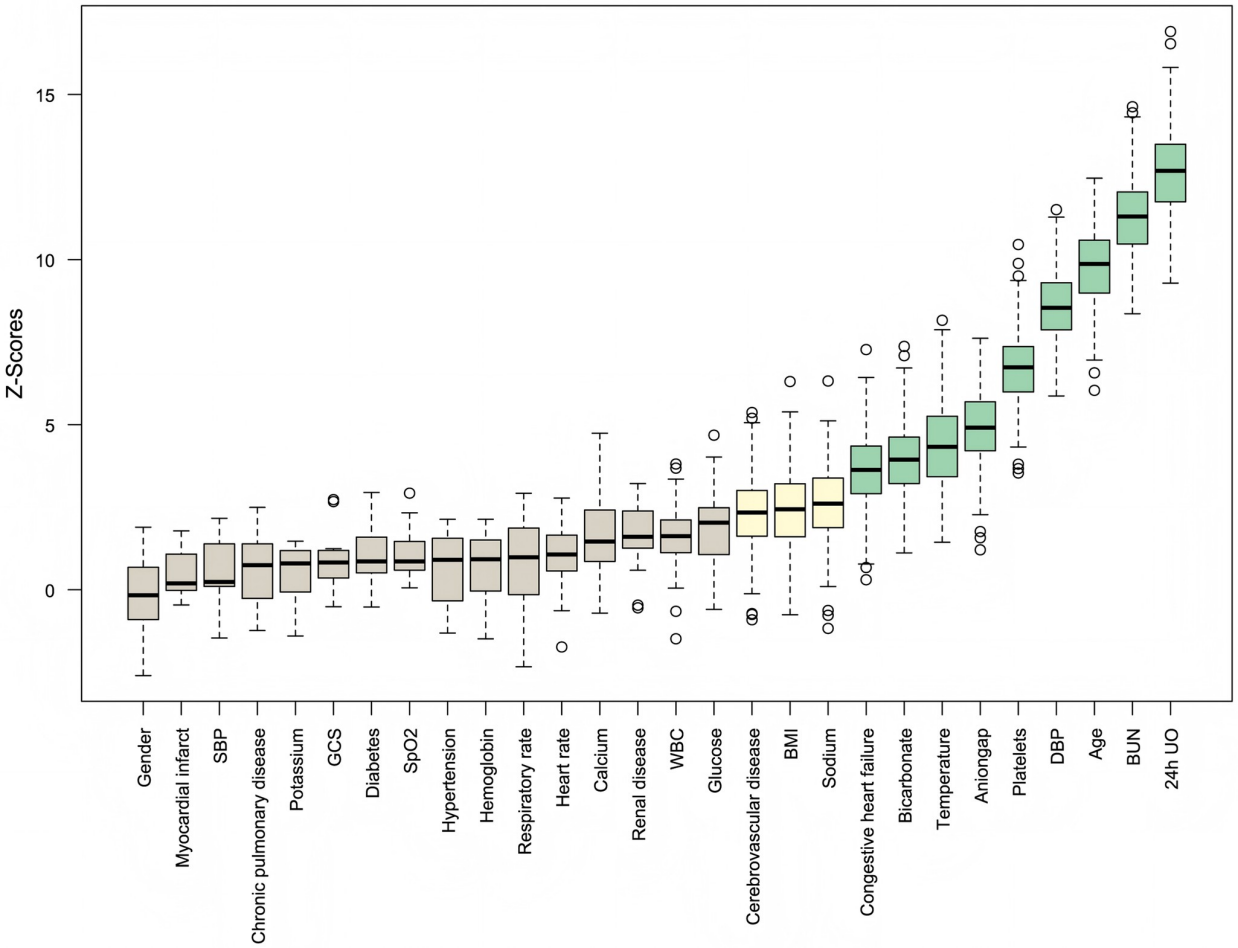
Feature selection results from the Boruta algorithm.

For the initially included 32 potential features, correlation analysis was performed first, and features with a correlation coefficient greater than 0.7 were excluded to avoid multicollinearity affecting model performance (S2 Fig). Then, all features were screened using the Boruta algorithm with 300 iterations, setting the confidence level at 0.01, and excluding features that were rejected, as shown in Fig 2.

### Model development

A stratified sampling strategy was used to randomly split the 2008–2019 data from the MIMIC database, with 80% used for training and 20% for validation. 14 ML models were constructed, including adaptive boosting (AdaBoost), category boosting (CatBoost), decision tree (DT), extra trees (ET), gradient boosting decision tree (GBDT), K-nearest neighbors (KNN), linear discriminant analysis (LDA), light gradient boosting machine (LightGBM), logistic regression (LR), multilayer perceptron (MLP), naive bayes (NB), random forest (RF), support vector machine (SVM), and eXtreme gradient boosting (XGboost). To optimize the prediction models and prevent overfitting, five-fold cross-validation and Bayesian search were used to obtain the final hyperparameters during model training (S3 Table). The trained ML models were then externally tested on the MIMIC database from 2020 to 2022.

The model performance was evaluated using metrics such as the area under the receiver operating characteristic (ROC) curve (AUC), accuracy, sensitivity, and specificity. The optimal cutoff value was determined by maximizing the Youden index (sensitivity + specificity—1). Calibration curves and decision curves were used to evaluate the calibration and clinical decision-making ability of the models. Furthermore, the clinical efficacy of the models was assessed by comparing their performance with commonly used clinical severity scoring systems.

### Feature selection and model explanation

SHAP is a method that calculates the contribution of input features to the final prediction and explains the decision-making process of the prediction model [13]. This approach includes both global and local explanations. The global explanation reveals the overall impact of each feature on the model, while the local explanation analyzes the contribution of individual sample features. The decision-making process of the final model was visualized using both global and local explanations provided by the SHAP method.

### Webpage deployment

To facilitate the use of the model in clinical settings, the final predictive model has been implemented and deployed into a web application built using the Streamlit Python library. When the operator provides the feature values required by the final model, the application can return an in-hospital mortality risk score for individual patients, along with a force plot illustrating the contribution of each feature for that patient.

### Statistical analysis

Data preprocessing, model development, performance evaluation, and result visualization were carried out using Python (version 3.9.18) and R (version 4.4.1). The Variance Inflation Factor (VIF) was utilized to assess potential multicollinearity among the 4 selected features (S4 Table).

Continuous variables with normal distribution were reported as means and standard deviations, while those not normally distributed were presented as medians (m) and interquartile ranges (IQR). Categorical variables were described using counts (n) and percentages (%). Differences in continuous variables were assessed using Student’s t-tests or Wilcoxon rank-sum tests, as appropriate. Categorical variables were analyzed with Chi-square tests or Fisher’s exact tests, depending on the data. A two-tailed p-value of less than 0.05 was considered statistically significant.

## Results

### Baseline characteristics

After screening according to the inclusion and exclusion criteria, a total of 1,315 patients from version 2.2 of the MIMIC-IV database and 579 patients from version 3.1 were identified (Fig 1). The baseline characteristics of all patients are shown in Table 1. In the overall study cohort, the median age was 64 [53–74] years, and 978 (65.2%) were male. In the derivation cohort, 318 patients died during hospitalization, with a mortality rate of 24.2%, while in the test cohort, 181 patients died during hospitalization, with a mortality rate of 31.3%. S2 Table provides an overview of the baseline characteristics of the survival and non-survival groups in the derivation cohort. Patients who died were older, had lower BMI, systolic blood pressure (SBP), diastolic blood pressure (DBP), mean blood pressure (MBP), and body temperature, as well as lower 24-hour urine output (UO). They also had a higher proportion of myocardial infarction, congestive heart failure, and renal disease, along with higher severity scores.

**Table 1:** Baseline characteristics of the derivation and test cohorts.

Variables	All	Derivation cohort	Test cohort	*P*-value
	(n = 1894)	(n = 1315)	(n = 579)	
**Demographics**				
Age, years	64 (53, 74)	65 (53, 75)	63 (52, 71)	0.004
Male, n(%)	1235 (65.2)	845 (64.3)	390 (67.4)	0.192
BMI	28.6 (24.5, 34.4)	28.0 (24.1, 33.7)	30.1 (25.6, 35.8)	<0.001
**Vital signs**				
Heart rate, bpm	85 (74, 96)	86 (76, 97)	82 (72, 95)	<0.001
SBP, mmHg	117 (108, 130)	118 (108, 132)	116 (107, 125)	<0.001
DBP, mmHg	59 (54, 67)	60 (54, 67)	59 (54, 65)	0.137
MBP, mmHg	77 (71, 84)	77 (71, 85)	76 (72, 83)	0.299
Respiratory rate, bpm	21 (18, 24)	20 (17, 23)	22 (18, 26)	<0.001
Temperature, °C	37.3 (36.9, 37.6)	37.3 (36.9, 37.7)	37.2 (36.8, 37.5)	<0.001
SpO2, %	97 (96, 99)	98 (96, 99)	97 (95, 98)	<0.001
24h UO, L	1.82 (1.02, 2.86)	1.82 (1.04, 2.81)	1.82 (0.96, 2.96)	0.948
**Laboratory tests**				
Hematocrit, %	28.8 (25.8, 32.8)	28.7 (25.8, 32.4)	29.0 (25.7, 34.3)	0.014
Hemoglobin, g/dL	9.5 (8.3, 10.8)	9.5 (8.4, 10.7)	9.4 (8.1, 11.0)	0.656
Platelets, K/μL	174 (110, 247)	162 (105, 232)	196 (125, 288)	<0.001
WBC, K/μL	11.2 (8.4, 14.9)	10.9 (8.2, 14.7)	11.8 (8.8, 15.6)	0.003
Aniongap, mmol/L	13 (10, 15)	13 (11, 16)	12 (10, 15)	<0.001
Bicarbonate, mmol/L	24 (22, 28)	24 (22, 27)	25 (22, 28)	0.082
Creatinine, mg/dL	1.1 (0.7, 2.0)	1.0 (0.7, 1.9)	1.2 (0.8, 2.2)	<0.001
BUN, mg/dL	26 (15, 43)	24 (14, 40)	30 (18, 49)	<0.001
Glucose, mg/dL	136 (114, 170)	132 (112, 163)	146 (120, 184)	<0.001
Sodium, mmol/L	140 (136, 144)	140 (137, 144)	140 (136, 144)	0.363
Potassium, mmol/L	4.0 (3.7, 4.4)	4.0 (3.7, 4.3)	4.1 (3.8, 4.5)	<0.001
Calcium, mmol/L	8.3 (7.9, 8.7)	8.2 (7.8, 8.6)	8.4 (8.0, 8.8)	<0.001
Chloride, mmol/L	104 (100, 109)	105 (101, 110)	102 (98, 107)	<0.001
**Clinical scores**				
SAPSII	42 (32, 53)	42 (32, 53)	42 (33, 53)	0.690
APSIII	53 (40, 71)	53 (40, 70)	53 (40, 71)	0.910
LODS	7 (5, 9)	7 (5, 9)	7 (5, 9)	0.003
OASIS	37 (32, 43)	37 (32, 43)	37 (32, 42)	0.615
GCS	15 (15, 15)	15 (15, 15)	15 (15, 15)	0.473
**Comorbidities, n (%)**				
Hypertension	735 (38.8)	533 (40.5)	202 (34.9)	0.020
Diabetes	626 (33.1)	400 (30.4)	226 (39.0)	<0.001
Myocardial infarct	361 (19.1)	238 (18.1)	123 (21.2)	0.108
Congestive heart failure	617 (32.6)	427 (32.5)	190 (32.8)	0.883
Cerebrovascular disease	474 (25.0)	342 (26.0)	132 (22.8)	0.137
Chronic pulmonary disease	528 (27.9)	383 (29.1)	145 (25.0)	0.068
Renal disease	423 (22.3)	285 (21.7)	138 (23.8)	0.298
**Other**				
Tracheotomy, n(%)	40 (2.1)	27 (2.1)	13 (2.3)	0.789

APS III, acute physiology and chronic health evaluation III; BMI, body mass index; BUN, blood urea nitrogen; DBP, diastolic blood pressure; GCS, Glasgow coma scale; LODS, logistic organ dysfunction system; MAP, mean arterial pressure; OASIS, Oxford acute severity of illness score; SAPS II, simplified acute physiology II; SBP, systolic blood pressure; SPO2, pulse blood oxygen saturation; UO, urine output; WBC, white blood cells.

### Model performance

**Fig 3:**
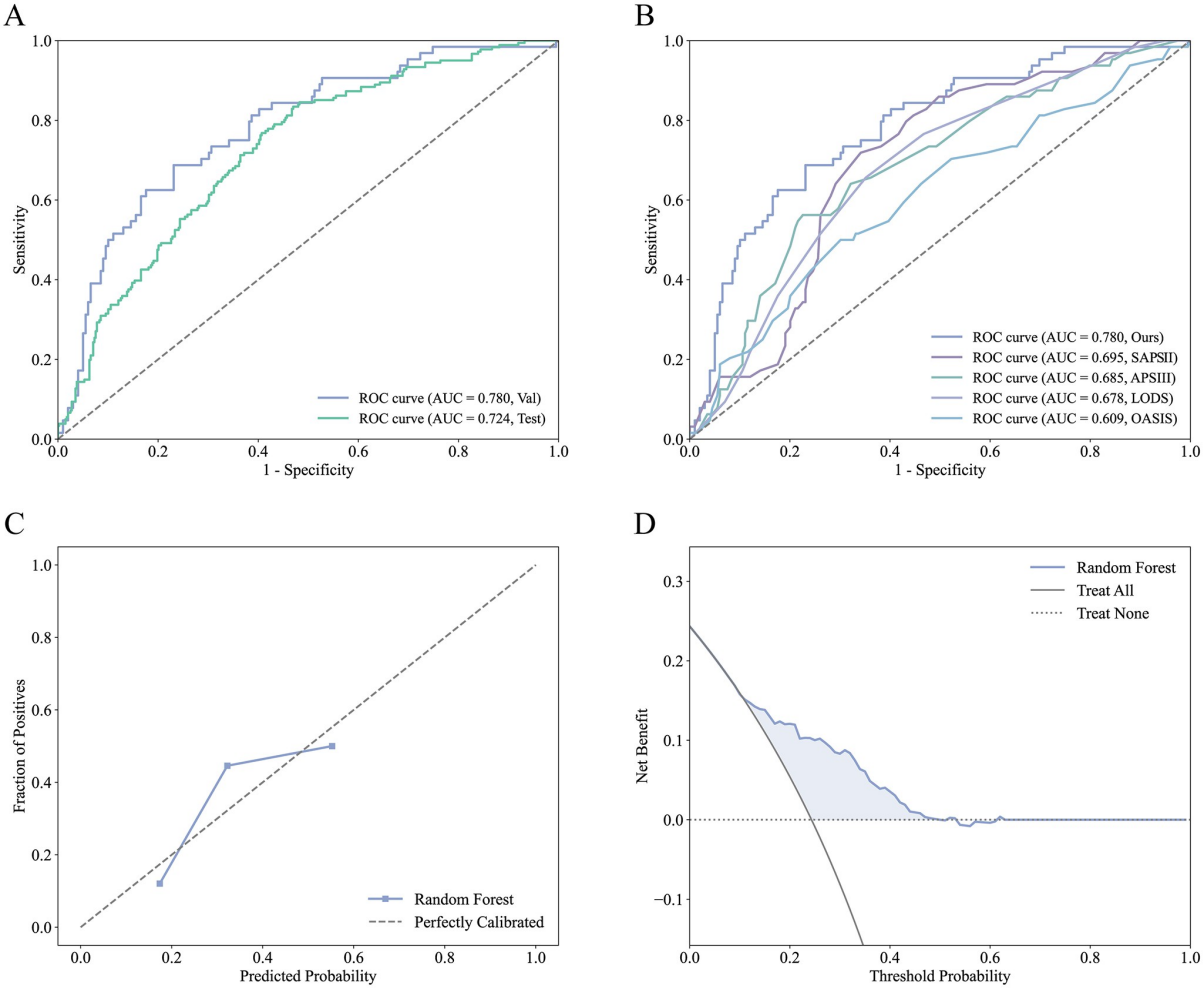
Performance visualization of the final random forest model. (A) ROC curves for the random forest model in both validation and test sets. (B) Comparison of the random forest model’s performance with clinical disease severity scores. (C) Calibration curve for the final model. (D) Decision curve analysis for the final model.

After feature selection, a total of 12 features were included in constructing the predictive model: 24h UO, BUN, age, DBP, platelets, anion gap, temperature, bicarbonate, congestive heart failure, sodium, BMI, and cerebrovascular disease, as shown in Fig 2. Among the 14 ML models developed, the CatBoost model demonstrated the best performance in the validation cohort with an AUC of 0.792 and an AUC of 0.709 in the test cohort. The RF model followed, with an AUC of 0.780 in the validation cohort and 0.724 in the test cohort (Table 2). After a comprehensive evaluation of all models in both the validation and test cohorts, the RF model was selected as the final model. Fig 3A, 3C and 3D show the ROC curve, calibration curve, and decision curve analysis (DCA) for the RF model. Fig 3B compares the performance of our final model with several commonly used clinical severity scoring systems, demonstrating that our model performed the best in this task.

**Table 2:** Performance of fourteen machine learning models in validation and test cohorts.

Models	Validation cohort				Test cohort			
	AUC	Accuracy	Sensitivity	Specificity	AUC	Accuracy	Sensitivity	Specificity
CatBoost	0.792	0.779	0.672	0.814	0.709	0.668	0.519	0.736
RF	0.780	0.749	0.688	0.769	0.724	0.670	0.608	0.698
LightGBM	0.771	0.734	0.750	0.729	0.703	0.674	0.624	0.696
ET	0.764	0.624	0.906	0.533	0.720	0.589	0.829	0.480
MLP	0.752	0.719	0.688	0.729	0.719	0.679	0.569	0.729
LDA	0.749	0.711	0.703	0.714	0.717	0.675	0.564	0.726
LR	0.745	0.741	0.625	0.779	0.722	0.698	0.497	0.789
GBDT	0.744	0.764	0.578	0.824	0.697	0.694	0.475	0.794
AdaBoost	0.741	0.802	0.516	0.894	0.693	0.705	0.359	0.862
NB	0.740	0.707	0.672	0.719	0.705	0.674	0.575	0.719
XGboost	0.740	0.684	0.719	0.673	0.706	0.656	0.641	0.663
DT	0.734	0.635	0.781	0.588	0.642	0.592	0.630	0.575
KNN	0.721	0.597	0.828	0.523	0.631	0.532	0.751	0.432
SVM	0.717	0.654	0.766	0.618	0.712	0.636	0.724	0.595

AdaBoost: adaptive boosting; AUC: area under the receiver-operating-characteristic curve; CatBoost: category boosting; DT: decision tree; ET: extra trees; GBDT: gradient boosting decision tree; KNN: K-nearest neighbors; LDA: linear discriminant analysis; LightGBM: light gradient boosting machine; LR: logistic regression; MLP: multilayer perceptron; NB: naive bayes; RF: random forest; SVM: support vector machine; XGboost: eXtreme gradient boosting.

### Model explanation

**Fig 4:**
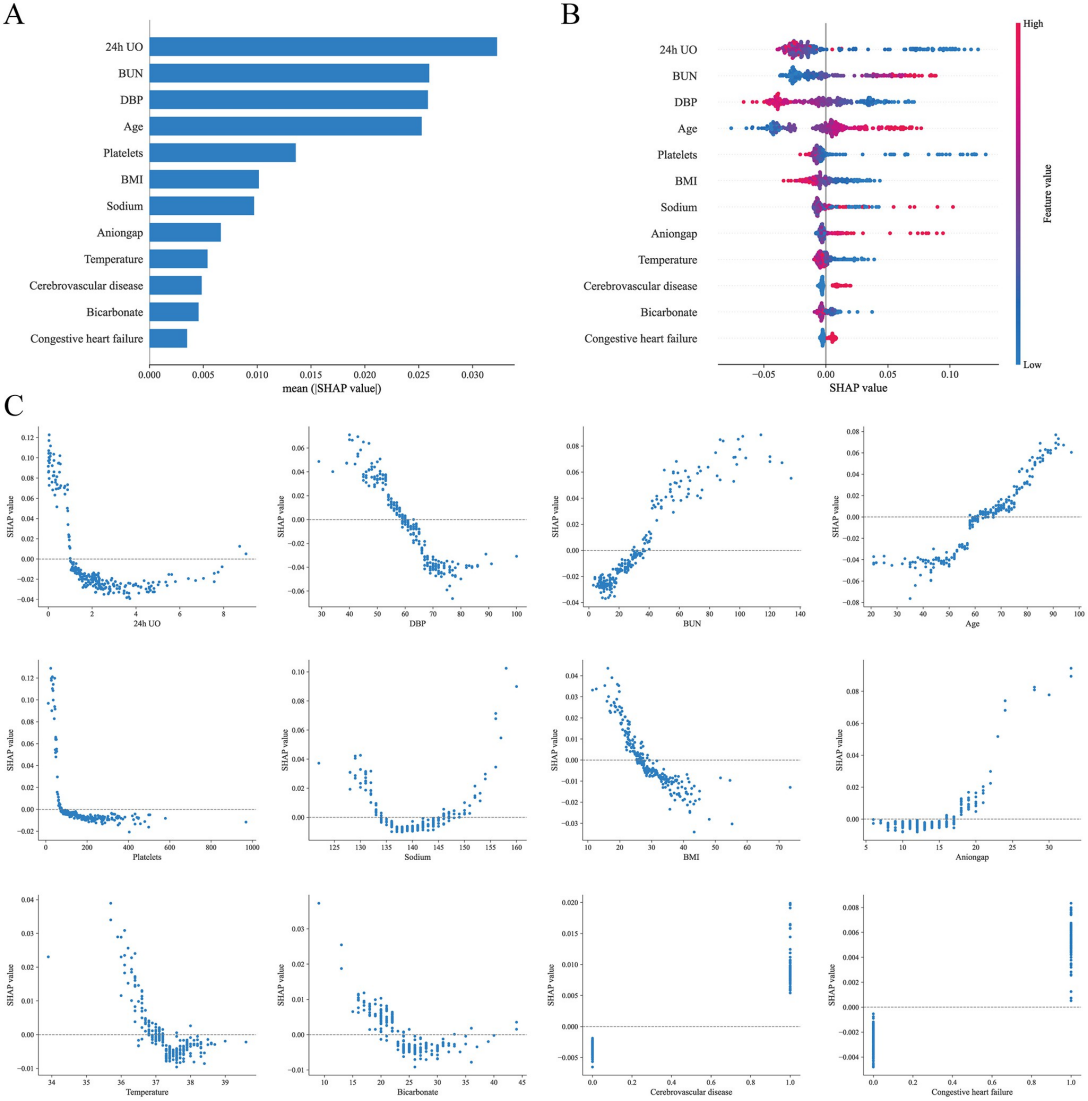
Global SHAP interpretation of the random forest model. (A) Global bar plot of SHAP values. (B) Global beeswarm plot of SHAP values. (C) Global dependence plots for individual features.

The SHAP method offers two types of explanations: global interpretation that analyze the model at the aggregate level, and local interpretation that focus on the individual level [14, 15]. The global interpretation is an overall description of the feature contributions. The SHAP summary plots for the RF model are displayed in Fig 4A and 4B, where the average SHAP values are used to assess the contributions of features to the model, sorted in descending order. Additionally, we further present the SHAP dependency plots for the 12 features, which facilitate a deeper understanding of how each feature contributes to the output of the RF model. As illustrated in Fig 4C, within the distribution of each feature’s values, SHAP values above zero contribute positively to the model’s prediction of in-hospital mortality risk for VAP patients, while SHAP values below zero contribute negatively.

**Fig 5:**
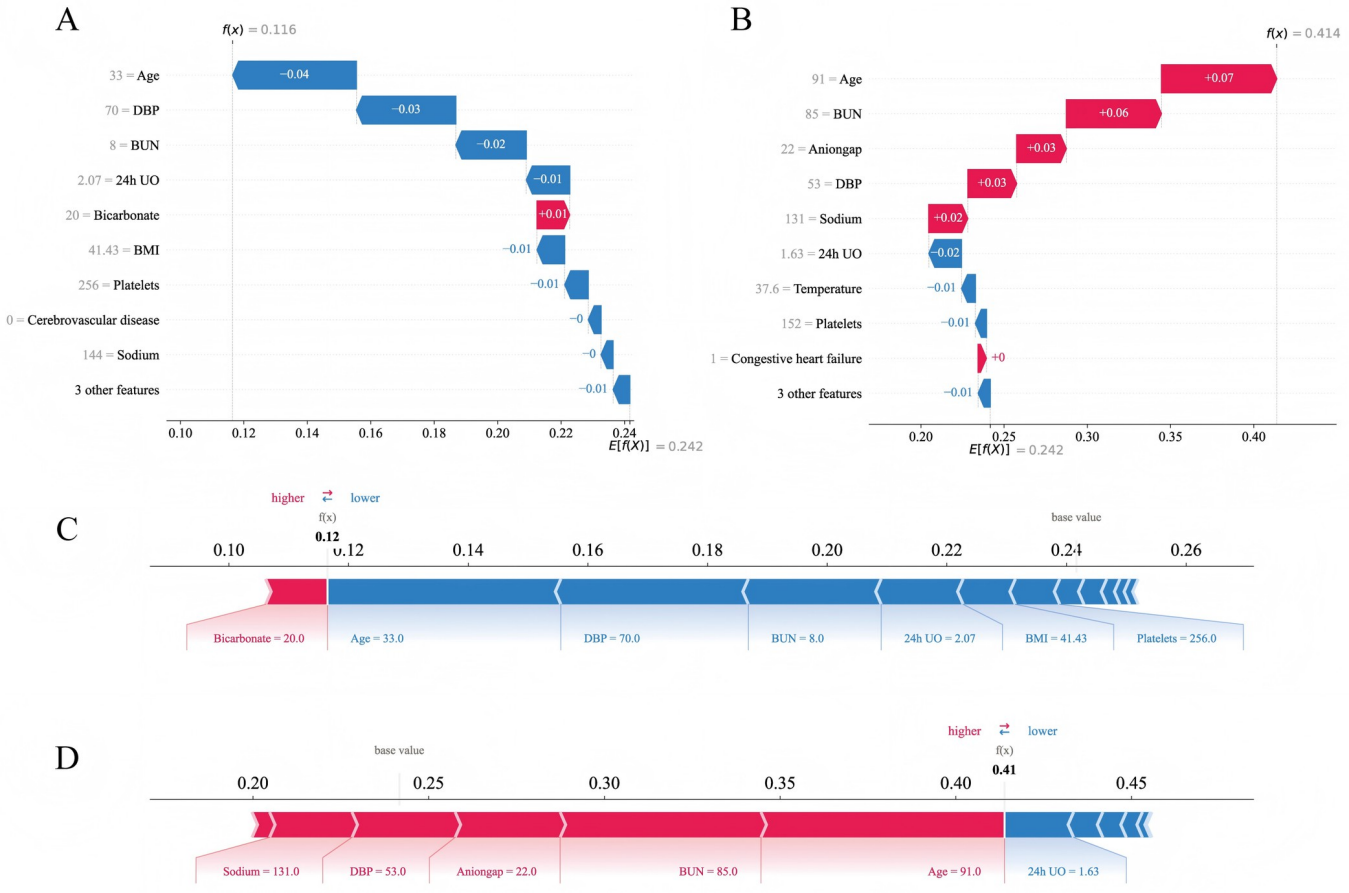
Local SHAP interpretations of the random forest model. (A) and (C) present waterfall and force plots for a low-risk case, while (B) and (D) present plots for a high-risk case. Red indicates a positive contribution of the feature to the outcome; blue indicates a negative contribution.

Moreover, local explanations analyze the contribution of each feature for a specific patient by inputting personalized data. We selected two representative samples for SHAP feature visualization analysis: one with actual in-hospital mortality and a high-risk prediction, and the other with actual survival and a low-risk prediction. As shown in Fig 5, for patients who died in the hospital, the SHAP analysis revealed that older age, higher BUN, anion gap, and sodium, as well as lower DBP, positively contributed to the model’s prediction of high risk, with age and BUN contributing the most to the prediction. Therefore, the model predicted this patient to be at high risk. For the patient who survived, SHAP analysis showed that younger age, relatively normal DBP, BUN, and 24-hour UO negatively contributed to the model’s prediction of high risk, leading the model to predict this patient as being at low risk.

### Application of the model

**Fig 6:**
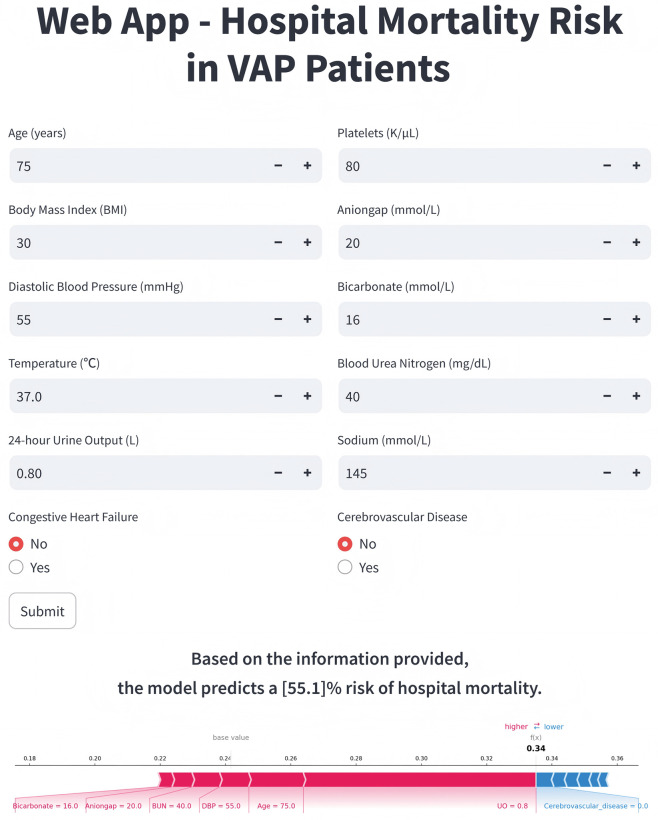
Visual representation of web application deployment.

When the actual values of the 12 features required by the model are input, the real-time web-based prediction tool automatically predicts the in-hospital mortality risk for an individual VAP patient (accessible at https://vaphospmortality.streamlit.app/). Fig 6 illustrates an example of using the application, where SHAP analysis force plot show that lower 24-hour UO, older age, relatively lower DBP and bicarbonate, as well as higher BUN and anion gap, all positively contributed to the model’s prediction of high risk for this patient. The model ultimately predicted the patient’s in-hospital mortality risk to be 59.6%.

## Discussion

In this retrospective study using a large public ICU database, a ML model was developed and validated based on clinical features, which was subsequently deployed as a web application to create an robust and clinically applicable prediction tool for assessing in-hospital mortality in VAP patients. Using the Boruta feature selection algorithm, this study successfully identified several key clinical features that significantly contribute to assessing in-hospital mortality, including 24-hour UO, BUN, DBP, age, platelets, BMI, among others. These features can be easily collected within one day after 48 hours of MV, making the model a promising early warning tool for predicting in-hospital mortality risk in VAP patients providing clinicians with an objective reference to guide further treatment. Additionally, this study assessed the generalizability of the model through external testing and demonstrated its clinical efficacy by comparing it to traditional scoring systems such as SAPS II and APS III.

In this study, 14 different ML algorithms were utilized to develop the prediction model, with the final results showing that the RF model exhibited the most stable and reliable performance. RF is an ensemble classifier that makes final decisions by constructing multiple decision trees and combining their predictions, and it is widely used as a classification model [16]. Numerous studies have shown that the RF method has excellent predictive value in the medical field. For example, a prospective multicenter cohort study by Hu et al. [17] evaluated data from critically ill children admitted to PICUs, using 11 machine learning algorithms to predict acute kidney injury (AKI) and outcomes, with RF demonstrating the best overall performance. Similarly, Chao et al. [18] included data from 555 patients with acute infections into seven models to predict 28-day mortality and found that the RF model outperformed other ML models in predicting mortality in infected patients.

Although ML algorithms have demonstrated good performance in various medical tasks, the "black box" nature of these models, which makes it difficult to interpret the prediction process, has been one of the barriers to clinical application [19]. SHAP, based on the game-theoretic concept of Shapley values, assigns each feature a contribution value, indicating its relative importance to the outcome [14]. Therefore, in this study, SHAP was employed to visualize the direction and magnitude of the influence of predictive factors on in-hospital mortality risk in VAP patients, thereby providing interpretability support for understanding the model’s decision-making process.

In the final prediction model, 24-hour UO had the highest importance in SHAP feature ranking. Low UO is an early marker of AKI and often indicates possible multi-organ dysfunction. Previous studies have consistently demonstrated that low UO is an important predictor of poor outcomes and mortality risk in ICU patients [20–22]. Moreover, low urine output reflects impaired circulatory function and hemodynamic instability, thereby increasing the risk of mortality, which aligns with another important feature in the model, DBP. BUN is an indicator of kidney function, and it significantly increases when the glomerular filtration rate decreases [23]. Elevated BUN also suggests a hypercatabolic state in patients, which is closely associated with disease progression. The study by Zhou et al. [24] and Wen et al. [22] showed that elevated BUN is one of the most important predictors of mortality risk in ICU patients, which is consistent with the findings of this study.

Platelet count and temperature were also important features in the model, often reflecting the severity of infection and inflammatory response. The results showed that thrombocytopenia and hypothermia were both associated with a higher risk of mortality. Thrombocytopenia suggests abnormalities in coagulation function and indicates an exacerbated systemic inflammatory response, whereas hypothermia may indicate a late stage of sepsis. Regarding admission age, older critically ill patients often have more chronic diseases and relatively weaker immune function, reducing their ability to fight infections. As indicated by this study, comorbid cerebrovascular disease and congestive heart failure increased the risk of in-hospital mortality in VAP patients. Additionally, several indicators of electrolyte imbalance and acid-base disturbances, such as sodium, anion gap, and bicarbonate, significantly impacted in-hospital mortality in our model, consistent with studies by Bales et al. [25] and Mao et al. [26].

In this study, BMI was also an important factor in predicting mortality risk in VAP patients. Traditionally, higher BMI is associated with an increased incidence of cardiovascular disease, diabetes, and other chronic conditions, which elevate mortality risk [27]. Interestingly, some critical care studies reveal an "obesity paradox" in the ICU setting [28–30], where higher BMI is associated with lower mortality in certain critically ill patient groups, consistent with the findings of this study. This paradox may be explained by the metabolic reserve that a higher BMI provides, enabling patients to better cope with infection and systemic inflammatory responses in the ICU. Overall, the impact of higher BMI on ICU mortality remains controversial and warrants further research.

Although the final model in this study exhibited notable reliability and generalizability in internal validation and external testing, the results suggest potential for further optimization. This may be attributable to the omission of infection- and immunology-related variables associated with VAP, which play critical roles in its onset, progression, and patient outcomes [4]. Previous studies have shown that cytokine storms in patients with COVID-19 and other severe infections are significantly associated with high mortality, particularly highlighting the pivotal roles of pro-inflammatory factors such as IL-6 and TNF-α in dysregulated inflammation [31]. In addition, VAP is typically caused by multiple pathogens, with the most prevalent being gram-negative bacteria (primarily Klebsiella, Acinetobacter, Pseudomonas, Escherichia coli, and other Enterobacteriaceae) and certain gram-positive species such as Staphylococcus aureus and Enterococci [32, 33]. Different pathogens may induce distinct inflammatory response patterns, resulting in diverse clinical outcomes. Rare pathogens responsible for VAP also warrant special attention. For instance, although Corynebacterium accolens is an uncommon pathogen, studies have confirmed its ability to cause ventilator-associated pneumonia [34].

Furthermore, the patient’s immune status is a key determinant in the course and prognosis of VAP. Research has indicated that heterogeneity in immune system function can significantly influence inflammatory responses and clinical outcomes [35]. For example, certain patients with primary immunodeficiency may exhibit heightened susceptibility to bacterial and viral infections due to defects in antiviral signaling pathways, exacerbating the progression of VAP.

To enhance the clinical applicability of our findings, a web-based application was developed based on the final RF model, developing a readily accessible online tool for predicting in-hospital mortality risk in VAP patients. This application aims to provide clinicians with a straightforward and intuitive interface, allowing them to effortlessly input relevant patient data and receive the model’s prediction, along with SHAP force plots that visualize the contributions of each feature. This facilitates more convenient use of our model by clinicians, with the potential for integration into practical clinical decision-making.

Several limitations exist in this study. First, this study is based on a retrospective design using an open-access database, which may lead to potential selection bias. Second, VAP patients were identified in the MIMIC-IV database using ICD codes, but due to the limitations of retrospective data, it is challenging to ascertain the exact timing of VAP diagnosis. To mitigate this issue, this study restricted the collection of clinical indicators to within 24 hours after 48 hours of MV, as VAP diagnosis typically requires at least 48 hours of ventilation. Third, this study did not include infection- and immunology-related factors associated with VAP, the inclusion of which may enhance the model’s predictive performance and clinical utility. Moreover, the complex and dynamic nature of ICU patients’ conditions was not fully accounted for, as this study did not further evaluate the impact of temporal changes in clinical characteristics of VAP patients during their ICU stay. Therefore, future multicenter prospective studies are warranted to better characterize the clinical course of VAP patients, validate these findings, and develop more accurate and reliable predictive models.

## Conclusion

This study developed and compared models to predict in-hospital mortality risk in VAP patients using various ML algorithms, among which the RF model demonstrated robust performance in both internal validation and external testing. The findings of this study indicate that factors such as UO, BUN, DBP, age, and platelet count are closely associated with in-hospital mortality in these patients. The ML model and online tool developed in this study have the potential to help clinicians effectively identify high-risk VAP patients, thereby reducing in-hospital mortality.

## Supporting information

S1 Dataset The derivation and test cohorts used in the study. (CSV)

S1 Fig The missing rate of each variable. (DOCX)

S2 Fig Heat map of correlation analyses among variables. (DOCX)

S1 Table The transparent reporting of a multivariable prediction model for individual prognosis or diagnosis—AI (TRIPOD—AI) checklist. (DOCX)

S2 Table Baseline characteristics between the survival and non-survival groups in the derivation cohort. (DOCX)

S3 Table Hyperparameters for 14 machine learning models. (DOCX)

S4 Table Covariance diagnosis for 12 features selected. (DOCX)

S5 Table Reasons for feature dropout in the derivation cohort. (DOCX)

## References

[1] Zimlichman E, Henderson D, Tamir O, Franz C, Song P, Yamin CK, et al. Health care-associated infections: a meta-analysis of costs and financial impact on the US health care system. JAMA Intern Med. 2013 Dec 9–23;173(22):2039–46. doi: 10.1001/jamainternmed.2013.9763 . 23999949

[2] Ramírez-Estrada S, Lagunes L, Peña-López Y, Vahedian-Azimi A, Nseir S, Arvaniti K, et al. Assessing predictive accuracy for outcomes of ventilator-associated events in an international cohort: the EUVAE study. Intensive Care Med. 2018 Aug;44(8):1212–1220. doi: 10.1007/s00134-018-5269-7 . 30003304 PMC7095084

[3] Larsson J, Itenov TS, Bestle MH. Risk prediction models for mortality in patients with ventilator-associated pneumonia: A systematic review and meta-analysis. J Crit Care. 2017 Feb;37:112–118. doi: 10.1016/j.jcrc.2016.09.003 . 27676171

[4] Howroyd F, Chacko C, MacDuff A, Gautam N, Pouchet B, Tunnicliffe B, et al. Ventilator-associated pneumonia: pathobiological heterogeneity and diagnostic challenges. Nat Commun. 2024 Jul 31;15(1):6447. doi: 10.1038/s41467-024-50805-z . 39085269 PMC11291905

[5] Li YT, Wang YC, Lee HL, Tsao SC, Lu MC, Yang SF. Monocyte Chemoattractant Protein-1, a Possible Biomarker of Multiorgan Failure and Mortality in Ventilator-Associated Pneumonia. Int J Mol Sci. 2019 May 6;20(9):2218. doi: 10.3390/ijms20092218 . 31064097 PMC6539645

[6] Pirracchio R, Cohen MJ, Malenica I, Cohen J, Chambaz A, Cannesson M, et al. Big data and targeted machine learning in action to assist medical decision in the ICU. Anaesth Crit Care Pain Med. 2019 Aug;38(4):377–384. doi: 10.1016/j.accpm.2018.09.008 . 30339893

[7] Ortiz-Barrios M, Arias-Fonseca S, Ishizaka A, Barbati M, Avendaño-Collante B, Navarro-Jiménez E. Artificial intelligence and discrete-event simulation for capacity management of intensive care units during the Covid-19 pandemic: A case study. J Bus Res. 2023 May;160:113806. doi: 10.1016/j.jbusres.2023.113806 . 36895308 PMC9981538

[8] Frondelius T, Atkova I, Miettunen J, Rello J, Vesty G, Chew HSJ, et al. Early prediction of ventilator-associated pneumonia with machine learning models: A systematic review and meta-analysis of prediction model performance✰. Eur J Intern Med. 2024 Mar;121:76–87. doi: 10.1016/j.ejim.2023.11.009 . 37981529

[9] Frondelius T, Atkova I, Miettunen J, Rello J, Jansson MM. Diagnostic and prognostic prediction models in ventilator-associated pneumonia: Systematic review and meta-analysis of prediction modelling studies. J Crit Care. 2022 Feb;67:44–56. doi: 10.1016/j.jcrc.2021.10.001 . 34673331

[10] Johnson AEW, Bulgarelli L, Pollard T, Gow B, Moody B, Horng S, et al. 2024. MIMIC-IV (version 3.1). PhysioNet. 10.13026/hxp0-hg59.

[11] Johnson AEW, Bulgarelli L, Shen L, Gayles A, Shammout A, Horng S, et al. MIMIC-IV, a freely accessible electronic health record dataset. Sci Data. 2023 Jan 3;10(1):1. doi: 10.1038/s41597-022-01899-x . 36596836 PMC9810617

[12] Collins GS, Moons KGM, Dhiman P, Riley RD, Beam AL, Van Calster B, et al. TRIPOD+AI statement: updated guidance for reporting clinical prediction models that use regression or machine learning methods. BMJ. 2024 Apr 16;385:e078378. doi: 10.1136/bmj-2023-078378 . 38626948 PMC11019967

[13] Scott M, Lundberg, Su-In Lee. 2017. A unified approach to interpreting model predictions. In Proceedings of the 31st International Conference on Neural Information Processing Systems (NIPS’17). Curran Associates Inc., Red Hook, NY, USA, 4768–4777.

[14] Lundberg SM, Erion G, Chen H, DeGrave A, Prutkin JM, Nair B, et al. From Local Explanations to Global Understanding with Explainable AI for Trees. Nat Mach Intell. 2020 Jan;2(1):56–67. doi: 10.1038/s42256-019-0138-9 . 32607472 PMC7326367

[15] Hu C, Li L, Huang W, Wu T, Xu Q, Liu J, et al. Interpretable Machine Learning for Early Prediction of Prognosis in Sepsis: A Discovery and Validation Study. Infect Dis Ther. 2022 Jun;11(3):1117–1132. doi: 10.1007/s40121-022-00628-6 . 35399146 PMC9124279

[16] Becker T, Rousseau AJ, Geubbelmans M, Burzykowski T, Valkenborg D. Decision trees and random forests. Am J Orthod Dentofacial Orthop. 2023 Dec;164(6):894–897. doi: 10.1016/j.ajodo.2023.09.011 . 38008491

[17] Hu J, Xu J, Li M, Jiang Z, Mao J, Feng L, et al. Identification and validation of an explainable prediction model of acute kidney injury with prognostic implications in critically ill children: a prospective multicenter cohort study. EClinicalMedicine. 2024 Jan 5;68:102409. doi: 10.1016/j.eclinm.2023.102409 . 38273888 PMC10809096

[18] Chao HY, Wu CC, Singh A, Shedd A, Wolfshohl J, Chou EH, et al. Using Machine Learning to Develop and Validate an In-Hospital Mortality Prediction Model for Patients with Suspected Sepsis. Biomedicines. 2022 Mar 29;10(4):802. doi: 10.3390/biomedicines10040802 . 35453552 PMC9030924

[19] Topol EJ. High-performance medicine: the convergence of human and artificial intelligence. Nat Med. 2019 Jan;25(1):44–56. doi: 10.1038/s41591-018-0300-7 . 30617339

[20] Yao RQ, Jin X, Wang GW, Yu Y, Wu GS, Zhu YB, et al. A Machine Learning-Based Prediction of Hospital Mortality in Patients With Postoperative Sepsis. Front Med (Lausanne). 2020 Aug 11;7:445. doi: 10.3389/fmed.2020.00445 . 32903618 PMC7438711

[21] Heffernan AJ, Judge S, Petrie SM, Godahewa R, Bergmeir C, Pilcher D, et al. Association Between Urine Output and Mortality in Critically Ill Patients: A Machine Learning Approach. Crit Care Med. 2022 Mar 1;50(3):e263–e271. doi: 10.1097/CCM.0000000000005310 . 34637423

[22] Wen C, Zhang X, Li Y, Xiao W, Hu Q, Lei X, et al. An interpretable machine learning model for predicting 28-day mortality in patients with sepsis-associated liver injury. PLoS One. 2024 May 20;19(5):e0303469. doi: 10.1371/journal.pone.0303469 . 38768153 PMC11104601

[23] Haines RW, Zolfaghari P, Wan Y, Pearse RM, Puthucheary Z, Prowle JR. Elevated urea-to-creatinine ratio provides a biochemical signature of muscle catabolism and persistent critical illness after major trauma. Intensive Care Med. 2019 Dec;45(12):1718–1731. doi: 10.1007/s00134-019-05760-5 . 31531715

[24] Zhou S, Lu Z, Liu Y, Wang M, Zhou W, Cui X, et al. Interpretable machine learning model for early prediction of 28-day mortality in ICU patients with sepsis-induced coagulopathy: development and validation. Eur J Med Res. 2024 Jan 3;29(1):14. doi: 10.1186/s40001-023-01593-7 . 38172962 PMC10763177

[25] Bales J, Cho S, Tran TK, Korab GA, Khandelwal N, Spiekerman CF, et al. The Effect of Hyponatremia and Sodium Variability on Outcomes in Adults with Aneurysmal Subarachnoid Hemorrhage. World Neurosurg. 2016 Dec;96:340–349. doi: 10.1016/j.wneu.2016.09.005 . 27637165

[26] Mao B, Ling L, Pan Y, Zhang R, Zheng W, Shen Y, et al. Machine learning for the prediction of in-hospital mortality in patients with spontaneous intracerebral hemorrhage in intensive care unit. Sci Rep. 2024 Jun 20;14(1):14195. doi: 10.1038/s41598-024-65128-8 . 38902304 PMC11190185

[27] Du Y, Lv Y, Zha W, Zhou N, Hong X. Association of body mass index (BMI) with critical COVID-19 and in-hospital mortality: A dose-response meta-analysis. Metabolism. 2021 Apr;117:154373. doi: 10.1016/j.metabol.2020.154373 . 32949592 PMC7493748

[28] Perez AV, Viana MV, Dall’Orto Thomazini L, Loss SH, de Machado FCR, do Nascimento AG, et al. BMI and mortality in critically ill patients with COVID-19: another brick in the wall of the obesity paradox. Obesity (Silver Spring). 2024 Aug;32(8):1474–1482. doi: 10.1002/oby.24069 . 38946013

[29] Xu D, Lu Y, Wang Y, Li F. The obesity paradox and 90 day mortality in chronic critically ill patients: a cohort study using a large clinical database. Eur J Med Res. 2024 Jul 29;29(1):392. doi: 10.1186/s40001-024-01962-w . 39075583 PMC11285416

[30] Dana R, Bannay A, Bourst P, Ziegler C, Losser MR, Gibot S, et al. Obesity and mortality in critically ill COVID-19 patients with respiratory failure. Int J Obes (Lond). 2021 Sep;45(9):2028–2037. doi: 10.1038/s41366-021-00872-9 . 34112941 PMC8190754

[31] Liu BM, Martins TB, Peterson LK, Hill HR. Clinical significance of measuring serum cytokine levels as inflammatory biomarkers in adult and pediatric COVID-19 cases: A review. Cytokine. 2021 Jun;142:155478. doi: 10.1016/j.cyto.2021.155478 . 33667962 PMC7901304

[32] Delle Rose D, Pezzotti P, Fortunato E, Sordillo P, Gini S, Boros S, et al. Clinical predictors and microbiology of ventilator-associated pneumonia in the intensive care unit: a retrospective analysis in six Italian hospitals. Eur J Clin Microbiol Infect Dis. 2016 Sep;35(9):1531–9. doi: 10.1007/s10096-016-2694-9 . 27272120

[33] Hellyer TP, Morris AC, McAuley DF, Walsh TS, Anderson NH, Singh S, et al. Diagnostic accuracy of pulmonary host inflammatory mediators in the exclusion of ventilator-acquired pneumonia. Thorax. 2015 Jan;70(1):41–7.doi: 10.1136/thoraxjnl-2014-205766 . 25298325 PMC4992819

[34] Liu BM, Beck EM, Fisher MA. The Brief Case: Ventilator-Associated Corynebacterium accolens Pneumonia in a Patient with Respiratory Failure Due to COVID-19. J Clin Microbiol. 2021 Aug 18;59(9):e0013721. doi: 10.1128/JCM.00137-21 . 34406882 PMC8372998

[35] Liu BM, Hill HR. Role of Host Immune and Inflammatory Responses in COVID-19 Cases with Underlying Primary Immunodeficiency: A Review. J Interferon Cytokine Res. 2020 Dec;40(12):549–554.doi: 10.1089/jir.2020.0210 . 33337932 PMC7757688

