# Supplementary figures and images for: An interpretable machine learning model for predicting in-hospital mortality in ICU patients with ventilator-associated pneumonia
Source: PLoS One. 2025 Jan 7;20(1):e0316526. doi: 10.1371/journal.pone.0316526 (PMC11706384; doi:10.1371/journal.pone.0316526)

**S1 Fig. The missing rate of each variable.**


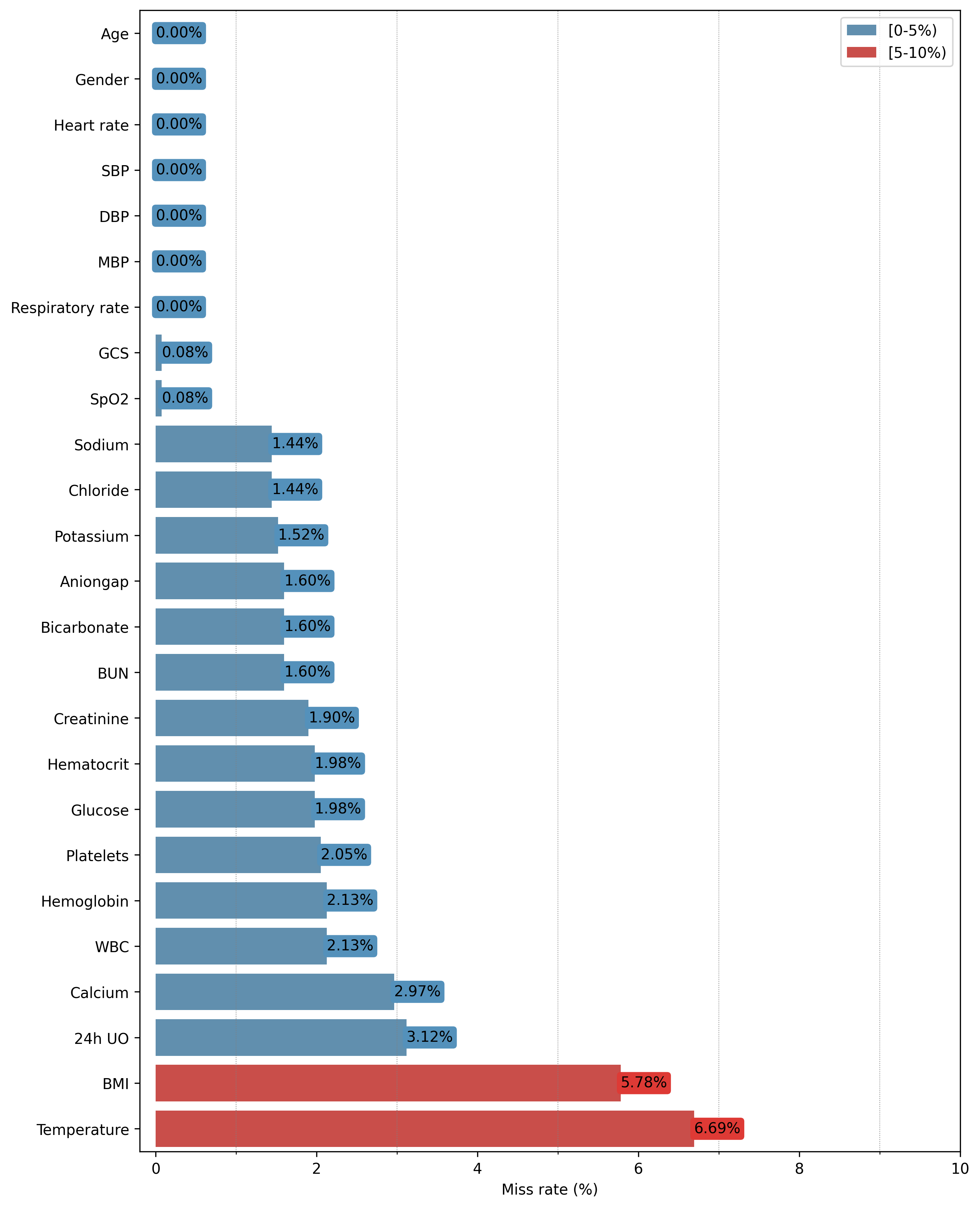

Supplement: S1 Fig — (DOCX) [file pone.0316526.s002.docx]

**S2 Fig. Heat map of correlation analyses among variables.**

**
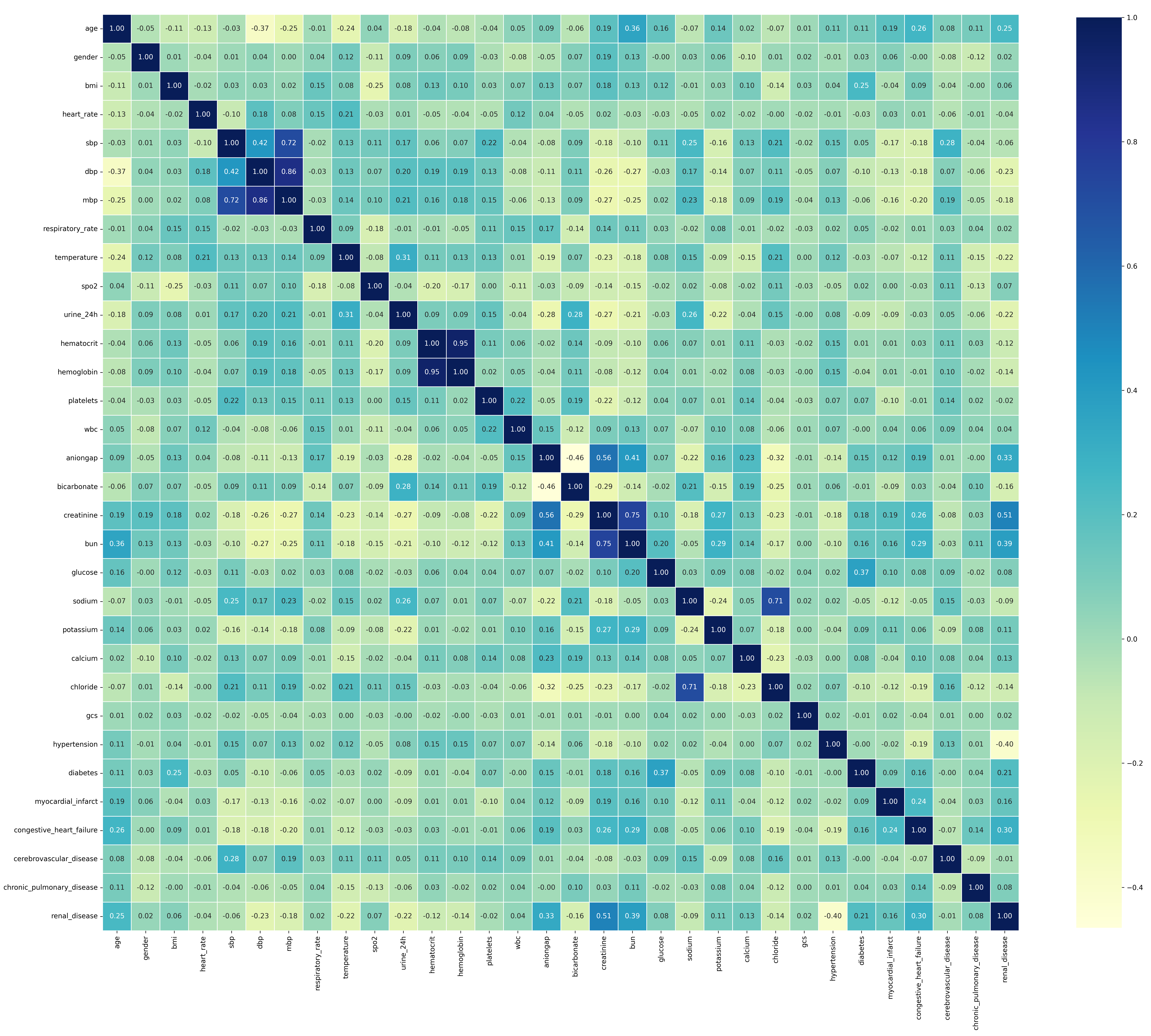
**

Supplement: S2 Fig — (DOCX) [file pone.0316526.s003.docx]
