# Supplementary material for: An interpretable machine learning model for predicting in-hospital mortality in ICU patients with ventilator-associated pneumonia
Source: PLoS One. 2025 Jan 7;20(1):e0316526. doi: 10.1371/journal.pone.0316526 (PMC11706384; doi:10.1371/journal.pone.0316526)
Supplement: S2 Table — (DOCX) [file pone.0316526.s005.docx]

| **Table S2. Baseline characteristics between the survival and non-survival groups in the derivation cohort.** | | | | |
| --- | --- | --- | --- | --- |
| **Variables** | **All  (n = 1315)** | **Survival  (n = 997)** | **Non-Survival  (n = 318)** | ***P-*value** |
|  |  |  |  |  |
| **Demographics** |  |  |  |  |
| Age, years | 65 (53, 75) | 63 (51, 73) | 71 (60, 80) | <0.001 |
| Male, n(%) | 845 (64.3) | 650 (65.2) | 195 (61.3) | 0.209 |
| BMI | 28.0 (24.1, 33.7) | 28.4 (24.3, 34.0) | 26.7 (23.1, 32.0) | 0.003 |
| **Vital signs** |  |  |  |  |
| Heart rate, bpm | 86 (76, 97) | 86 (75, 97) | 87 (77, 98) | 0.256 |
| SBP, mmHg | 118 (108, 132) | 120 (109, 133) | 114 (107, 129) | <0.001 |
| DBP, mmHg | 60 (54, 67) | 61 (55, 68) | 57 (50, 63) | <0.001 |
| MBP, mmHg | 77 (71, 85) | 78 (72, 86) | 74 (69, 81) | <0.001 |
| Respiratory rate, bpm | 20 (17, 23) | 20 (17, 23) | 21 (18, 24) | 0.027 |
| Temperature, ℃ | 37.3 (36.9, 37.7) | 37.3 (37.0, 37.7) | 37.1 (36.7, 37.6) | <0.001 |
| SpO2, % | 98 (96, 99) | 98 (96, 99) | 97 (96, 99) | 0.164 |
| 24h UO, L | 1.82 (1.04, 2.81) | 1.96 (1.19, 2.97) | 1.30 (0.65, 2.11) | <0.001 |
| **Laboratory tests** |  |  |  |  |
| Hematocrit, % | 28.7 (25.8, 32.4) | 28.7 (25.9, 32.5) | 28.4 (25.7, 31.8) | 0.194 |
| Hemoglobin, g/dL | 9.5 (8.4, 10.7) | 9.5 (8.5, 10.7) | 9.4 (8.3, 10.6) | 0.075 |
| Platelets, K/μL | 162 (105, 232) | 168 (109, 235) | 150 (86, 220) | <0.001 |
| WBC, K/μL | 10.9 (8.2, 14.7) | 10.7 (8.2, 14.4) | 11.8 (8.3, 16.1) | 0.049 |
| Aniongap, mmol/L | 13 (11, 16) | 13 (11, 15) | 14 (11, 17) | <0.001 |
| Bicarbonate, mmol/L | 24 (22, 27) | 25 (22, 28) | 23 (21, 26) | <0.001 |
| Creatinine, mg/dL | 1.0 (0.7, 1.9) | 0.9 (0.7, 1.6) | 1.4 (0.8, 2.4) | <0.001 |
| BUN, mg/dL | 24 (14, 40) | 22 (13, 35) | 30 (18, 54) | <0.001 |
| Glucose, mg/dL | 132 (112, 163) | 130 (112, 159) | 138 (115, 172) | 0.008 |
| Sodium, mmol/L | 140 (137, 144) | 140 (137, 144) | 140 (135, 144) | 0.107 |
| Potassium, mmol/L | 4.0 (3.7, 4.3) | 3.9 (3.7, 4.3) | 4.1 (3.7, 4.4) | 0.003 |
| Calcium, mmol/L | 8.2 (7.8, 8.6) | 8.2 (7.8, 8.6) | 8.2 (7.9, 8.7) | 0.158 |
| Chloride, mmol/L | 105 (101, 110) | 106 (101, 110) | 105 (100, 109) | 0.111 |
| **Clinical scores** |  |  |  |  |
| SAPS II | 42 (32, 53) | 40 (31, 50) | 48 (40, 57) | <0.001 |
| APS III | 53 (40, 70) | 52 (39, 67) | 61 (47, 81) | <0.001 |
| LODS | 7 (5, 9) | 6 (5, 9) | 8 (6, 10) | <0.001 |
| OASIS | 37 (32, 43) | 37 (32, 42) | 39 (34, 45) | <0.001 |
| GCS | 15 (15, 15) | 15 (15, 15) | 15 (15, 15) | 0.364 |
| **Comorbidities, n (%)** |  |  |  |  |
| Hypertension | 533 (40.5) | 419 (42.0) | 114 (35.9) | 0.051 |
| Diabetes | 400 (30.4) | 293 (29.4) | 107 (33.7) | 0.151 |
| Myocardial infarct | 238 (18.1) | 163 (16.4) | 75 (23.6) | 0.004 |
| Congestive heart failure | 427 (32.5) | 295 (29.6) | 132 (41.5) | <0.001 |
| Cerebrovascular disease | 342 (26.0) | 246 (24.7) | 96 (30.2) | 0.051 |
| Chronic pulmonary disease | 383 (29.1) | 277 (27.8) | 106 (33.3) | 0.058 |
| Renal disease | 285 (21.7) | 191 (19.2) | 94 (29.6) | <0.001 |
| **Other** |  |  |  |  |
| Tracheotomy, n(%) | 27 (2.1) | 27 (2.7) | 0 (0.0) | 0.003 |
| APS III, acute physiology and chronic health evaluation III; BMI, body mass index; BUN, blood urea nitrogen; DBP, diastolic blood pressure; GCS, Glasgow coma scale; LODS, logistic organ dysfunction system; MAP, mean arterial pressure; OASIS, Oxford acute severity of illness score; SAPS II, simplified acute physiology II; SBP, systolic blood pressure; SPO2, pulse blood oxygen saturation; UO, urine output; WBC, white blood cells. | | | | |
