# Supplementary material for: An interpretable machine learning model for predicting in-hospital mortality in ICU patients with ventilator-associated pneumonia
Source: PLoS One. 2025 Jan 7;20(1):e0316526. doi: 10.1371/journal.pone.0316526 (PMC11706384; doi:10.1371/journal.pone.0316526)
Supplement: S3 Table — (DOCX) [file pone.0316526.s006.docx]

| **Table S3**. **Hyperparameters for 14 machine learning models.** | |
| --- | --- |
| **Models** | **Hyperparameters** |
| CatBoost | n_estimators, 30; learning_rate, 0.005; max_depth, 4 |
| RF | n_estimators, 139; max_depth, 4; max_features, sqrt |
| LightGBM | n_estimators, 125; learning_rate, 0.021; max_depth, 3 |
| ET | n_estimators, 500; max_depth, 6; max_features, sqrt |
| MLP | solver, lbfgs; max_iter, 991; activation, identity |
| LDA | tol, 1.341; shrinkage, 0.0003; solver, lsqr |
| LR | C, 0.273; max_iter, 5000; penalty, l2; solver, liblinear |
| GBDT | n_estimators, 176; learning_rate, 0.006; max_depth, 3 |
| AdaBoost | n_estimators, 50; learning_rate, 0.043; algorithm, SAMME.R |
| NB | var_smoothing, 0.001 |
| XGboost | n_estimators, 50; learning_rate, 0.015; max_depth, 3 |
| DT | criterion, entropy; max_depth, 4; max_features, sqrt |
| KNN | n_neighbors, 45; algorithm, kd_tree; weights, distance |
| SVM | C,0.073; kernel, linear |
| AdaBoost: adaptive boosting; CatBoost: category boosting; DT: decision tree; ET: extra trees; GBDT: gradient boosting decision tree; KNN: K-nearest neighbors; LDA: linear discriminant analysis; LightGBM: light gradient boosting machine; LR: logistic regression; MLP: multilayer perceptron; NB: naive bayes; RF: random forest; SVM: support vector machine; XGboost: eXtreme gradient boosting. | |
