# Supplementary material for: An interpretable machine learning model for predicting in-hospital mortality in ICU patients with ventilator-associated pneumonia
Source: PLoS One. 2025 Jan 7;20(1):e0316526. doi: 10.1371/journal.pone.0316526 (PMC11706384; doi:10.1371/journal.pone.0316526)
Supplement: S4 Table — (DOCX) [file pone.0316526.s007.docx]

| **Table S4**. **Covariance diagnosis for 12 features selected.** | |
| --- | --- |
| **Features** | **VIF** |
| Age | 1.350 |
| BMI | 1.096 |
| DBP | 1.215 |
| Temperature | 1.161 |
| 24h UO | 1.224 |
| Platelets | 1.067 |
| Aniongap | 1.824 |
| Bicarbonate | 1.501 |
| BUN | 1.436 |
| Sodium | 1.150 |
| Congestive heart failure | 1.173 |
| Cerebrovascular | 1.096 |
| BMI, body mass index; BUN, blood urea nitrogen; DBP, diastolic blood pressure; UO, urine output; VIF, variance inflation factor. | |
