# Supplementary material for: An interpretable machine learning model for predicting in-hospital mortality in ICU patients with ventilator-associated pneumonia
Source: PLoS One. 2025 Jan 7;20(1):e0316526. doi: 10.1371/journal.pone.0316526 (PMC11706384; doi:10.1371/journal.pone.0316526)
Supplement: S5 Table — (DOCX) [file pone.0316526.s008.docx]

| **Table S5. Reasons for feature dropout in the derivation cohort.** | |
| --- | --- |
| **Reasons for feature dropout** | **Feature name** |
| Missing rate > 20% | PO2, PCO2, PH, Lactate, Albumin, INR, PT, PTT, ALT, AST, Bilirubin, CRP |
| Correlation coefficient > 0.7 | MBP, Hematocrit, Creatinine, Chloride |
| Boruta algorithm | Glucose, WBC, Renal disease, Calcium, Heart rate, Respiratory rate, Hemoglobin, Hypertension, SpO2, Diabetes, GCS, Potassium, Chronic pulmonary disease, SBP, Myocardial infarct, Gender |
